# Simple and promising paper-based electrochemical platform for serological detection of American tegumentary leishmaniasis

**DOI:** 10.1590/0074-02760230149

**Published:** 2024-02-12

**Authors:** Daniela E Barraza, Paula I Nanni, María E Bracamonte, Roberto E Chaile, Carla B Goy, Leonardo Acuña, Jorge D Marco, Rossana E Madrid

**Affiliations:** 1 Consejo Nacional de Investigaciones Científicas y Técnicas, Universidad Nacional de Tucumán, Facultad de Ciencias Exactas y Tecnología, Instituto Superior de Investigaciones Biológicas, Laboratorio de Medios e Interfases, Tucumán, Argentina; 2 Consejo Nacional de Investigaciones Científicas y Técnicas, Universidad Nacional de Salta, Facultad de Ciencias de la Salud, Instituto de Patología Experimental, Salta, Argentina

**Keywords:** leishmaniasis, electrochemistry, graphite electrodes, paper-based, impedance, neglected disease, immunosensor

## Abstract

**BACKGROUND:**

American tegumentary leishmaniasis (ATL) is an endemic neglected tropical disease (NTD), its conventional treatment is toxic, slow, and invasive. Rapid diagnosis is crucial for the clinical management of suspected patients, so the development and use of low-cost, miniaturised and portable devices could be the key.

**OBJECTIVES:**

This work aimed to develop a simple paper-based electrochemical platform for the serological detection of ATL.

**METHODS:**

Platform was fabricated in Whatman N°1 paper, contains a hydrophobic zone generated by wax printing, two pencil graphite electrodes, and uses specific crude extracts (CA) antigens for ATL immuno-determination. The platform performance was analysed by measuring the relative impedance change for different antigen-antibody combinations. Then, 10 serum human samples previously diagnosed by the gold standard (five positive ATL cases and five non-ATL cases) were evaluated.

**FINDINGS:**

The platform presented a linear response for the charge transfer resistance (ΔRct) and the interface reactance (ΔXc). Also, optimal working conditions were established (1/60 serum dilution and 180 µg/mL CA concentration). Then, the platform permits to distinguish between ATL and non-ATL (p < 0.05) human serum samples.

**MAIN CONCLUSIONS:**

Our platform could allow the diagnosis, management, and monitoring of leishmaniasis while being an extremely simple and environmentally friendly technology.

Leishmaniasis is a group of diseases caused by the flagellate protozoan of the *Leishmania* genus and it is classified according to different clinical manifestations, such as tegumentary (cutaneous or mucosal) and visceral manifestations.^1^ Health agencies classify it as a neglected tropical disease (NTD),^2^ because they are neglected and defunded by the global health agenda and financing agents. However, NTDs have a higher prevalence in tropical and subtropical regions, especially in places with difficult access, internal conflicts, poverty, and low visibility on the part of the government, even though the most affected population requires attention from the government public health system. In addition, it is associated with stigmatisation and social exclusion.^2^ ^,^ ^3^ Thus, the cycle of poor educational outcomes and limited career opportunities is perpetuated. Therefore, the World Health Organization (WHO) recommends its research in several aspects, especially regarding diagnostic tools, drugs, and vaccines.^1^

In Argentina, American tegumentary leishmaniasis (ATL) is endemic and must be reported.^4^ Of the approximately 30 species that are part of the *Leishmania* genus,^5^ *Leishmania (Viannia) braziliensis* has been implicated in 90% of ATL cases in our country.^6^ ^,^ ^7^ ^,^ ^8^ Conventional treatment for this disease is relatively toxic, slow, invasive, and expensive, and although this clinical form is not fatal, it causes considerable morbidity in patients;^1^ therefore, a rapid diagnosis is crucial for the patient.

Currently, various methods are used to diagnose leishmaniasis. The parasitological method, consisting of microscopically searching for amastigotes in smears and culturing promastigotes, is the most frequently used method to diagnose ATL in public health systems in endemic countries. However, this method has some intrinsic limitations: amastigote detection in smears is invasive and has a low sensitivity, particularly in ATL, which requires trained personnel and is time consuming.^6^ ^,^ ^7^ ^,^ ^8^ ^,^ ^9^ ^,^ ^10^ ^,^ ^11^ On the other hand, molecular techniques based on polymerase chain reaction (PCR) are not routinely used because of their complexity and difficulty in being implemented in clinical practice, despite presenting good diagnostic performance (high sensitivity and specificity).

In contrast to the previously mentioned diagnostic techniques, serological methods, such as enzyme-linked immunosorbent assay (ELISA), have greater versatility because they offer the possibility of analysing many samples with high reproducibility and low cost. Currently, it is commonly used to diagnose visceral leishmaniasis (VL), and despite having a higher sensitivity for the diagnosis of ATL than rudimentary parasitological tests, it is still not used for routine diagnosis because of the lack of optimised conditions and/or standards for this technique.^12^ ^,^ ^13^

Bracamonte et al.^6^ performed for the first time a method to diagnose ATL by means of ELISA with a membrane of crude antigens (MCA-ELISA), with very good results. Crude antigenic extracts (CA) were obtained from promastigotes and amastigotes of macrophage cultures of *L. (V.) braziliensis*, the causative agent of ATL in our region.

These results are encouraging to carry out seroprevalence studies or to monitor the efficacy of the treatments carried out by the patients. However, the need for inputs (secondary antibodies, plastic material, etc.), equipment (plate shakers, plate readers, etc.), and trained personnel makes it difficult to carry out the test in remote areas, among other things.

A crucial aspect in the control of endemic leishmaniasis is the development of sensitive, specific, and suitable diagnostic systems for the socioeconomic conditions in which this disease develops.^6^ In this context, and as happened in the recent coronavirus disease 2019 (COVID-19) pandemic, the development and use of low-cost, miniaturised, and easy-to-use devices, such as biosensors, is the key for providing rapid disease diagnosis.^14^ ^,^ ^15^ Within the diversity of biosensors that can be developed and found in the market, electrochemical biosensors are distinguished by presenting a wide variety of techniques based on the specificity of each system (voltammetry, amperometry, potentiometry, impedancemetry, etc.). In addition, the simultaneous determination of different analytes and analysis in complex samples is possible without requiring expensive equipment.^14^ ^,^ ^16^

Currently, in the sensor industry, as in others, there is a great demand for the development, manufacture, and application of eco-friendly processes to reach a sustainable future with a minimal environmental impact. So, the objective is to implement simple analytical methodologies that have a low sample consumption, low waste and cost being fast, feasible for nonspecialised personnel, and easy to automate. Additionally, miniaturisation is another important factor, in order to incorporate the sensors in relevant devices for different application areas, such as clinical, food, and environmental.^17^ ^,^ ^18^ ^,^ ^19^

Paper-based electrochemical biosensors have become attractive analytical devices owing to their eco-friendly features, such as disposability, incinerability, and biodegradability. Other important characteristics are their portability, simple and inexpensive manufacturing processes, easy functionalisation and surface modification, and the possibility of simple immobilisation of reagents in the areas of interest. In addition, fluid circulation in these devices can occur passively via capillarity; therefore, the use of pumps for fluid transport is not necessary.^16^ ^,^ ^20^ ^,^ ^21^ ^,^ ^22^ These characteristics make them suitable for deployment in resource-constrained settings such as emerging economies or field environments.^22^

In addition, pencil graphite electrodes (PGE) have gained popularity in various electrochemical applications^23^ and are frequently used in research and industrial settings because of their unique properties, including low cost, good electrical conductivity, chemical stability, commercial availability, and reusable quality.^24^ Graphite, the main component of these electrodes, is abundantly available and relatively inexpensive compared to other electrode materials, such as platinum or gold.^25^ This property makes PEGs suitable for a wide range of applications, including electroanalysis, electrochemical sensing, and energy storage systems. PGEs exhibit remarkable chemical stability, making them resistant to oxidation and corrosion in many electrolytes.^26^ This stability allows for long-term and repetitive use without significant degradation of electrode performance. Considering that paper-based electrochemical platforms are excellent examples of green sensors.^18^

The objective of this work was to develop a paper-based microfluidic platform that allows serological detection of antibodies against ATL. The platform includes PGE, made with pencil lead, to carry out immunodetection through electrochemical measurements. The proposed biosensor is a simple and eco-friendly economic solution for the future detection of ATL.

## MATERIALS AND METHODS

*Equipment, reagents, and biomolecules* - Electrochemical measurements were performed at room temperature (25ºC) using a Solartron 12508 W system (Solartron Analytical, Hampshire, UK) composed of a Solartron 1287 electrochemical analyser and a Solartron 1250 frequency response analyser, commanded by the software provided by the manufacturer (ZPlot^®^ and CorrWare^®^, Scribner Associates Incorporated, North Carolina, USA). An electronic connector with gold contacts was used to connect the electrochemical cells to the measuring equipment. In addition, a humid chamber system was used for each measurement to avoid evaporation of the samples.

The substance used as the electron transfer mediator for electrochemical measurements was a potassium ferrocyanide/potassium ferricyanide pair: K_4_ [Fe (CN)_6_] 3H_2_O/K_3_ [Fe (CN)_6_] (Fe^+2^ / Fe^+3^) (Sigma Aldrich - St. Louis, MO, USA) 1mM, which was prepared in a KH_2_PO_4_/Na_2_HPO_4_ 0.01 M buffer solution (PBS) at pH 7.2 with 0.1 M KCl as the supporting electrolyte (Cicarelli TM - Santa Fe, Argentina).

Biological samples such as anti-*Leishmania* antibodies (AB), sera from ATL human cases, positive (+), n = 5; healthy individuals or no ATL cases, negative (-), n = 5; and CA extracts were provided by the Experimental Pathology Institute (IPE) - Salta, Argentina, according to Bracamonte,^6^ with the permission and signed consent of all patients and donors who voluntarily agreed to participate anonymously in this study.

**Fig. 1:**
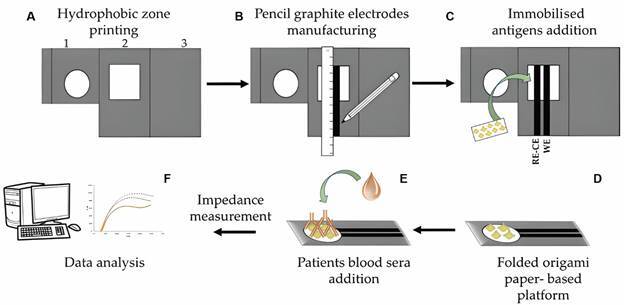
paper-based electrochemical platform for serological detection of American tegumentary leishmaniasis (ATL). Platform preparation steps and mechanism of detection are as follows: (A) Hydrophobic zone printing. In grey are shown the printed hydrophobic areas. The details of the three folding sections of the device are: section 1 for sample placement, section 2 of the electrochemical cell, where the nitrocellulose paper will be placed, and section 3 is a complete hydrophobic support area. (B) Pencil graphite electrodes manufacturing. The working electrode (WE) and the reference electrode-counter electrode (RE-CE) were constructed manually using a ruler until resistances of about 1 kΩ or less were obtained. (C) Immobilised antigens addition. Nitrocellulose paper with *Leishmania (Viannia) braziliensis* CA extract inmobilisated is placed on bipolar pencil graphite electrodes (PGE) cell. (D) Folded origami paper- based platform ready for measurements. (E) Patients blood sera addition. The scheme represents the addition of a positive serum sample for ATL, to the platform, leading to the formation of the antigen-antibody complex. (F) At the end, after impedance measurement using a Solartron 12508 W system, data analysis can be performed.

*Electrochemical microfluidic paper-based device manufacturing* - The proposed device has an origami structure (Fig. 1) fabricated using Whatman N°1 chromatographic paper (Whatman International Ltd.). It contains a hydrophobic zone, which was generated using a wax printing technique using a solid ink printer (Xerox Colorqube 8580, Spain). Once the hydrophobic zone (dark grey colour) was printed, the wax was melted on a hot plate set at 110ºC for 5 min to impregnate the entire paper thickness.

As shown in Fig. 1, the device is folded into three parts (sections 1, 2 and 3) along the dotted lines. Section 1 contains a 7 mm-diameter circle to place the sample. Section 2 contains the bipolar cell with PGE fabricated with a pencil lead (separation distance between electrodes = 1 mm) and a hydrophilic 10 x 10 mm^2^ zone, which is intended for antigen localisation. Finally, section 3 is a completely hydrophobic zone that prevents solution leakage.

*Manufacturing and evaluation of pencil graphite electrodes* - As previously mentioned, PGE fabrication was carried out using pencil leads. The pencils for their manufacture were selected according to the European Letter Scale, where graphite pencils are named with a letter, H (hardness) or B (blackness), and a number that indicates the degree of hardness or blackness. Type B leads contain more graphite and are softer than the type H leads. For this reason, the pencils selected for this work have B leads, which contain a greater amount of graphite, the conductive element necessary for manufacturing the electrodes. Foster et al.^27^ analysed several pencils for the fabrication of PEGs and found that 6B exhibited the largest current peak and that 5B showed the best electrochemical reversibility. Therefore, in this work, these two types of pencils were studied. Commercial pencils of grade 5B (Faber-Castell TM, Germany) and 6B (Lyra TM, Germany), obtained from a local shop, were used. The working electrode (WE) and the reference electrode-counter electrode (RE-CE) were constructed manually. Whatman N° 1 paper was placed on a hard marble surface, and approximately 40 pencil strokes were made using a ruler until resistances on the order of 1 kΩ or less were obtained. The resistance was measured using a multimeter (Uyigao Technology Co., Ltd.). Electrochemical and hardness evaluations were performed to determine whether the chemical composition of the pencil leads contributed to their electrochemical performance. For electrochemical characterisation, cyclic voltammetry and impedance measurements were performed in triplicate using a Solartron system with a 1 mM Fe^+2^/Fe^+3^ solution. Voltammograms were obtained at scan rate 50 mV.s^-1^ (-900 to 900 mV potential range), and the cathodic (Ipc) and anodic (Ipa) peak currents of each one were used to compare the cells. Impedance data were obtained with 0V polarisation potential and applying an AC potential of 7.1 mV amplitude at a frequency range of 0.1 Hz to 65 kHz. Scanning electron microscopy (SEM) was performed using a Zeiss Supra 55 VP (Carl Zeiss SMT, Oberkochen, Germany) scanning electron microscope (Centro Integral de Microscopía Electrónica (CIME), UNT-CONICET) to evaluate hardness leads.

*Immobilisation of the bioreceptor* - Five different *L. (V.) braziliensis* CA extract concentrations (0.018 µg/mL, 0.18 µg/mL, 1.8 µg/mL, 18 µg/mL, and 180 µg/mL) and a control without CA extract were prepared by dilution in PBST buffer solution (0.01 M PBS with 0.1 M KCl pH 7.3 and 0.05% Tween 20).

Next, an aliquot of 5 µL of each CA extract dilution was placed on 7 x 10 mm^2^ nitrocellulose paper (Protran, Cytiva, Sweden) and allowed to dry for 10 min.

They were then incubated with 700 µL of blocking buffer (PBS buffer added at 1% with skimmed milk (Nestlé Sbelty)) for 1 h at room temperature with gentle agitation. This step is necessary to block the remaining active sites and avoid possible nonspecific binding of the proteins present in the sera of the patients to be analysed. Finally, excess blocking buffer was removed, with 700 µL of PBS shaken gently for 3 min. The washing procedure was repeated three times with excess removal after each wash. Then, nitrocellulose paper with immobilised CA extracts was dried and stored for 24 h at 4ºC until the electrochemical measurements.

*Electrochemical evaluation of the immunoreaction* - To determine the optimal concentrations of CA extracts for immobilisation and anti-*L. (V.) braziliensis* antibodies for the electrochemical immunosensor, a double titration assay was performed using the paper-based microfluidic device. For this, dilutions in Fe^+2^ / Fe^+3^ solution (1/180, 1/60, 1/20 and 1/6.7) of pooled positive (+) and negative (-) blood sera were used.

The CA-AB interaction was analysed by impedance measurements for each CA extract concentration in combination with each anti-*L. (V.) braziliensis* AB dilution in triplicate and in a particular device for each measurement. As reaction controls, measurements of Fe^+2^/Fe^+3^ without sera solution and PBS without antigens were performed.

From the Argand diagrams, the interface reactance (Xc) and charge transfer resistance (Rct) values were obtained by fitting the experimental data to a semicircle using Zview software. Because the width of each electrode depends on the width of each lead, the normalised values of Xc and Rct were obtained for the surface of each electrode for each type of pencil used. The parameters analysed were the variation in ∆Rct [Ω] = (Rct1-Rct2) and ∆Xc [Ω] = (Xc1 – Xc2) (Xc: interface reactance) before (1) and after (2) the addition of (+) and (-) serum pools with respect to the blank (Fe^+2^/Fe^+3^).

*Immunogenicity study* - The preparation of the paper-based platform and mechanism of detection were displayed in Fig. 1. Ten real human blood serum samples, previously diagnosed by gold standard lab techniques at IPE, were evaluated using the paper-based platform by impedance measurements: five positive ATL cases (+) and five non-ATL cases (-). The optimal working combination CA-AB determined from the previous assay was used for the measurements. For these tests, 10 µL of each diluted blood serum [(+) or (-)] was placed on the surface of the immunosensor, and all measurements were performed in triplicate. Immunoreactions between the immobilised CA extracts and anti-*L. (V.) braziliensis* AB were studied by measuring the variations in Rct and Xc before and after the addition of the (+) or (-) serum samples. A paper-based microfluidic device was used for each test. In addition, the percentage variation of ∆Rct [%] = [(Rct1-Rct2)/Rct1] x 100 and ∆Xc [%] = [(Xc1 – Xc2)/Xc1] x 100 was calculated.

*Statistical data analyses* - To assess whether there was a significant difference between the analysed parameters ∆Rct and ∆Xc for positive and negative sera, a t test was used to analyse the normality of the sample. The results were considered statistically significant at p < 0.05.

*Ethics statement* - The procedures and informed consent forms signed by the subjects were approved by the Bioethics Committee of the Medical College of Salta and the Bioethics Committee of the Health Ministry of Salta, Argentina, following the principles of the Declaration of Helsinki. The last re-evaluation of projects, including the diagnosis of leishmaniasis, was approved on 19 June 2019 under number 321, Resolution 136934/2018-0.

## RESULTS

*Evaluation of pencil graphite electrodes* - Two types of electrochemical cells were fabricated with 2-mm wide PGE electrodes: one using a 5B lead and the other using a 6B lead. In terms of conductivity, both types of PGE electrodes achieved electrical resistance values below 1 kΩ.

**Fig. 2:**
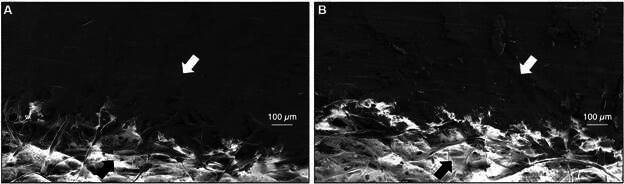
scanning electron microscopy images of the pencil graphite electrodes (PGE). The white arrows indicate the electrode surface, and the black arrows indicate the paper surface. (A) PGE was traced using a 5 B pencil. (B) PGE was traced using 6 B pencil. Magnification 200X.

Figure 2A and B shows electron microscopy images of the two types of electrode surfaces fabricated with pencils 5B and 6B, respectively. In both cases, abrasion of the paper produced by the pressure of the pencil strokes is seen as a difference in height between the electrode (paper fibres fully covered with graphite) and the paper. The surface of the electrodes made with a harder pencil (5B) has a smooth morphology, while less hard pencils, such as 6B, give rise to irregular surface electrodes with noticeable graphite structures.

**Fig. 3:**
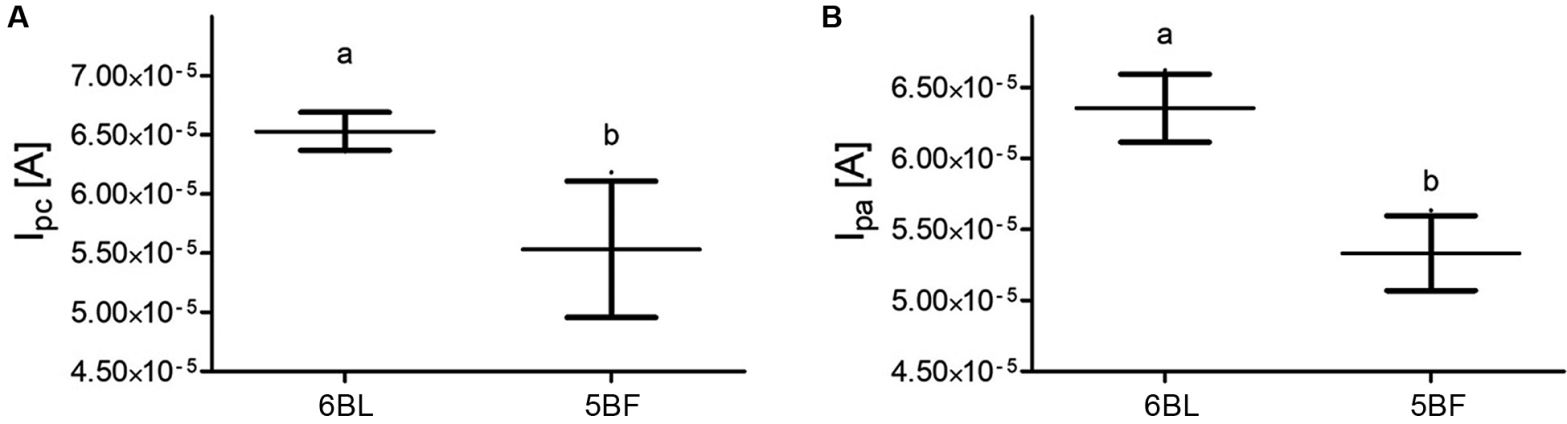
values of (A) cathodic (Ipc) and (B) anodic (Ipa) peak currents of each voltammogram obtained with the cells based on each of the analysed pencils (X-axis). The measurements were performed in Fe^+2^ / Fe^+3^ 1 mM redox solution, at a scan rate of 50 mV.s^-1^. Values of triplicate measurements are represented by their mean (central line) and standard deviation (whiskers). Different letters indicate significant differences (p < 0.05).

**Fig. 4:**
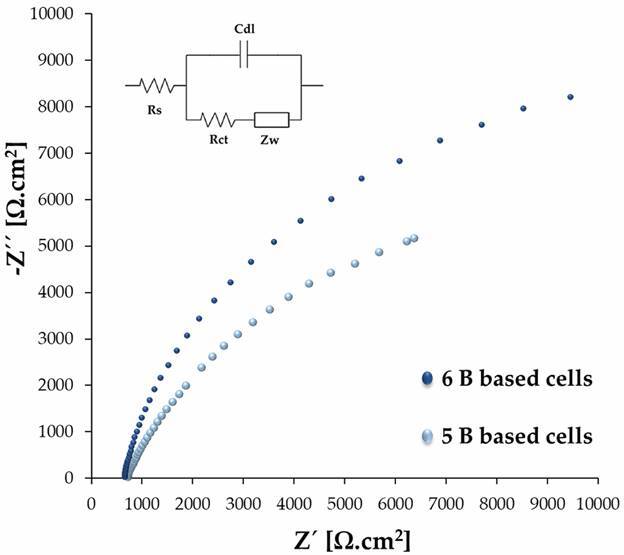
argand diagram (Z’’ [Ω.cm^2^] vs. Z’ [Ω.cm^2^]) of paper-based microfluidic cells made with 6 B and 5 B graphite electrodes. Impedance data were obtained in Fe^+2^ / Fe^+3^ 1 mM redox solution, with 0V polarisation potential, and applying an AC potential of 7.1 mV amplitude at a frequency range of 0.1 Hz to 65 KHz. The insert is the equivalent circuit applied to model impedance spectra data in the presence of the redox solution, double layer capacitance (Cdl), solution resistance (Rs), the charge-transfer resistance (Rct) and Warburg Impedance (Zw).

From the voltammograms (scan rate 50 mV.s^-1^), obtained for each cell type, analysis of the cathodic (Ipc) and anodic (Ipa) current peaks was performed (Fig. 3A-B, respectively). The 6B mine-based cells presented the highest mean Ipc and Ipa and a lower standard deviation compared with the values obtained for 5B mine-based cells. The former indicates that the redox process is beneficial in this type of cell, and the latter implies a higher repeatability of the fabrication process, which is very important for sensor fabrication. The Argand diagram (Fig. 4) allows the evaluation of the impedance behaviour of different PGEs, normalised with respect to the electrode area. Although the cells based on mine 6B have higher impedance than those based on mine 5B, the difference is not as high, and the Rct values are similar; thus, considering the benefit of reproducibility and higher currents, cells based on mine 6B were selected for use in the development of the immunosensor device.

**Fig. 5:**
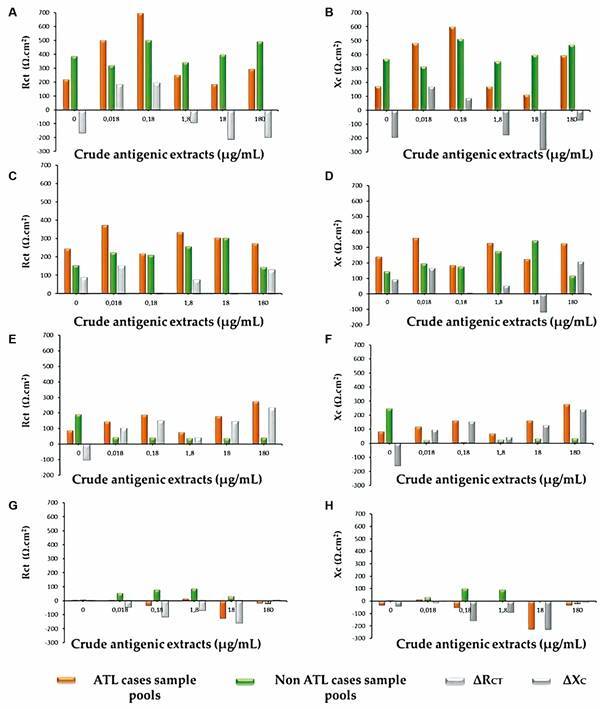
charge transfer resistance (Rct.Ω cm^2^) and interface reactance (Xc.Ω cm^2^) values for each CA extract concentration (0.018, 0.18, 1.8, 18, and 180 µg/mL) in combination with the different serum pool dilutions 1/6.7 (A and B), 1/20 (C and D), 1/60 (E and F), and 1/180 (G and H) for American tegumentary leishmaniasis (ATL) (+) and non-ATL (-). The corresponding ΔRct and ΔXc values of the (+) and (-) samples are depicted in grey.

*Electrochemical detection of the immunoreaction* - Fig. 5 shows Rct [Ω.cm^2^] and Xc [Ω.cm^2^] values for each CA extract concentration (0.018, 0.18, 1.8, 18, and 180 µg/mL) in combination with the different serum pool dilutions for ATL and non-ATL cases, 1/6.7 (A and B), 1/20 (C and D), 1/60 (E and F) and 1/180 (G and H), obtained from the Argand diagram applying the fit circle. The corresponding ΔRct and ΔXc values of the difference between the Rct and Xc values for (+) and (-) sera are also depicted.

**Fig. 6:**
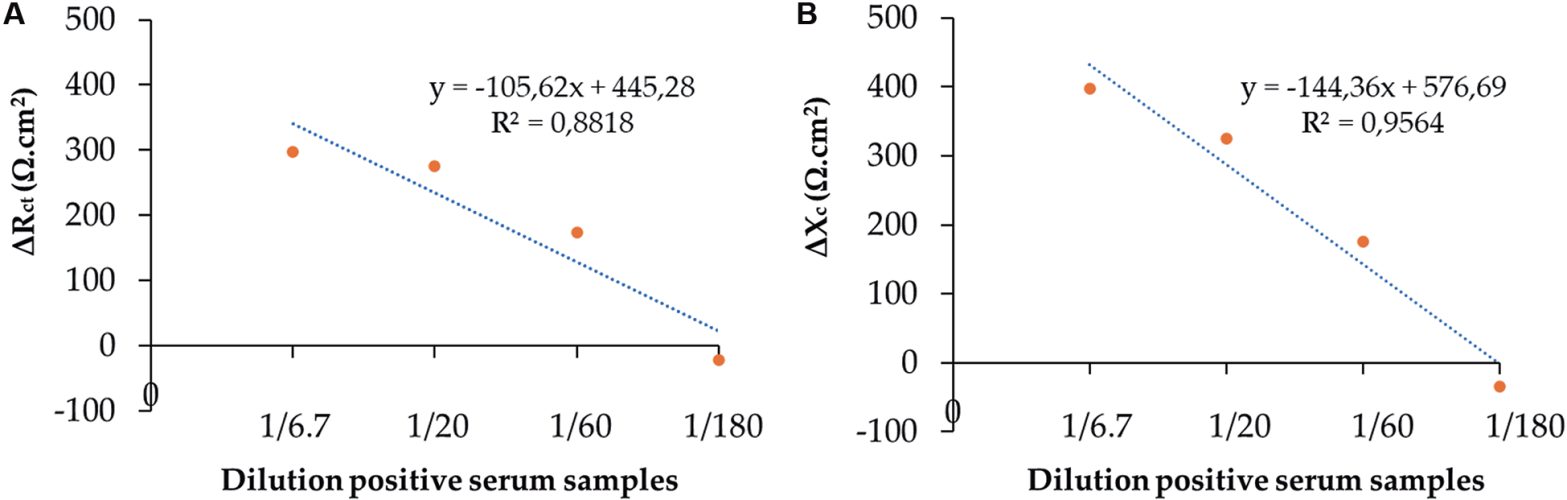
correlation curves for (A) charge transfer resistance variation (ΔRct.Ω cm^2^) and (B) Interface reactance variation (ΔXc.Ω cm^2^) as a function of the positive serum pool dilution factor.

As seen in Fig. 5, cases E and F, corresponding to 1/60 dilution of sera, show the greatest difference in Rct and Xc values between the positive and negative serum pools. On the other hand, the highest difference was observed for the cells containing immobilised CA extract at a concentration of 180 µg/mL. Fig. 6 shows the correlation curve between the dilutions evaluated and their combination with this concentration of CA extract. The values of both parameters decreased as the ATL-positive antibody sample was diluted. Both charge transfer resistance and interface reactance showed a linear relationship with diluted serum samples (R^2^ = 0.8818) (A) and (R^2^ = 0.9564) (B), respectively. Considering these results, a combination of 1/60 serum dilution and CA extract concentration of 180 µg/mL was selected for the next immunoassay test.

**Fig. 7:**
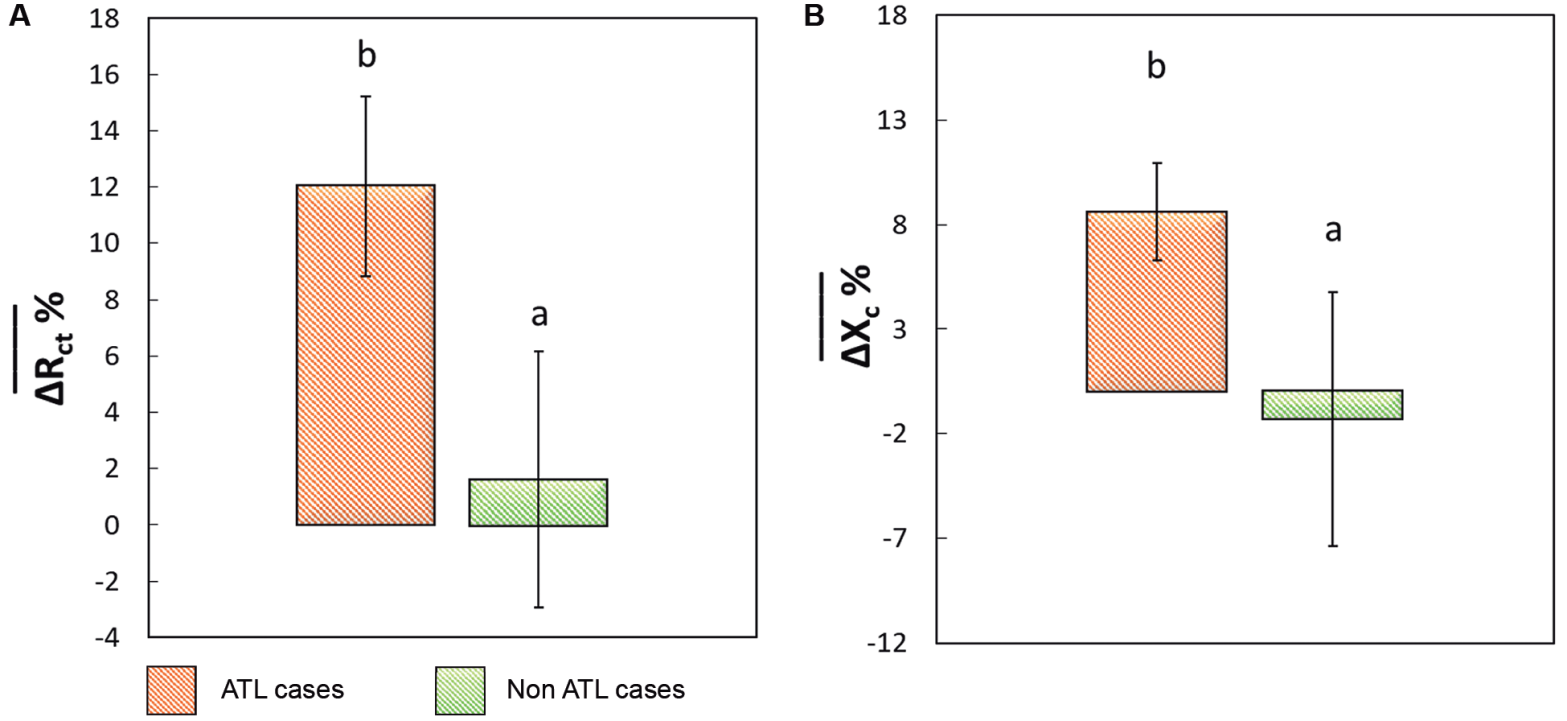
histogram of (A) the variation in the normalised charge transfer resistance (∆Rct%) and (B) the variation in the normalised interface reactance (∆Xc%) for American tegumentary leishmaniasis (ATL) cases and non-ATL cases with the developed paper-based microfluidic platform. Different letters indicate significant differences (p < 0.05).

*Paper-based electrochemical platform performance* - A small population of patients was evaluated impedancimetrically using a simple platform as a proof of concept. Fig. 7 shows the histograms of the ∆Rct% (A) and ∆Xc% (B) values for the positive and negative sera analysed, with their means and standard deviations. The analysis of the parameters ∆Rct% and ∆Xc% showed significant differences (p < 0.05) between both groups.

## DISCUSSION

This paper presents for the first time a simple device that uses paper-based microfluidic technology with electrochemical measurements for immunodetection of ATL.

In particular, our device combines a previously developed specific high-throughput diagnostic method for ATL,^6^ the use of cellulose and nitrocellulose [increasingly popular support materials for the fabrication of paper-based microfluidic chips or microfluidic paper-based analytical devices (µPAD)],^16^ ^,^ ^28^ the use of PGE and electrochemistry. This combination gives rise to an extremely simple and sustainable technology platform,^29^ with the potential to provide a solution for the diagnosis of an NTD as ATL.

In the present work, typical commercially available pencils were used to manufacture PGE with very good results. However, unlike other works,^30^ the selection of the type of pencil and the number of abrasions were crucial for the fabrication and performance of the selected PGE. In this case, we demonstrate that the Lyra 6B pencil exhibits the best electrochemical performance for the manufacture of paper electrodes in terms of hardness, conductivity, and acceptable resistance to charge transfer. In addition, the cells proposed here, which have paper as a substrate and PGEs, are entirely biodegradable, fulfilling the principle of the cradle-to-cradle philosophy. Thus, at the end of their lives, they can return to Earth and are completely safe.^31^ This is another advantage of the developed cells.

One of the limitations of this type of electrode is the low reproducibility of the manual manufacturing process,^32^ which could result in low repeatability of the measurements. However, this can be addressed through automatised graphite pencil stroking devices, such as those presented by Rao et al.^33^ and Jayapiriya and Sanket,^34^ which reduce user intervention, variability, and fabrication time. In addition, in some cases, as presented in this work, relative measurements can be performed to eliminate variability among cells.

Through impedance measurements, it was established that the analysed parameters ∆Rct and ∆Xc in patients with ATL were significantly higher than those in healthy patients. These results are in agreement with those reported in another work^35^ in which the Rct values for serum samples from dogs with VL were significantly higher than those in non-VL cases. This difference could be due to the formation of the AC-AB complex, which leads to an increase in the circuit impedance.

As mentioned above, the problems faced by these neglected diseases lead to a scarcity of research on them, as well as the availability of rapid diagnostic devices in the market.^14^

Different technologies such as immunosensors and genosensors have been proposed for the diagnosis of leishmaniasis.^36^ However, within these varieties, we have only found a few attempts for the immunodiagnosis of cutaneous leishmaniasis. These studies are summarised and compared with our platform, in Table. For example, for the immunodetection of cutaneous leishmaniasis,^37^ a biosensor system made with nanostructured films containing specific CAs of *L.* (*Leishmania*) *amazonensis* and *Trypanosoma cruzi* was developed using capacitance measurements as detection method. The unprecedented selectivity of this device was possible because of the antigen-antibody molecular recognition processes inherent to the detection with immobilised antigens and the statistical correlation of the electrical impedance data, which allowed them to distinguish between real samples positive for Chagas and leishmaniasis disease. The biosensor could distinguish between blood sera samples containing 10^-5^ mg/mL of antibody solution within a few minutes. A laborious technique is used to construct biosensors and gold interdigitated electrodes. Instead, our device uses simple graphite electrodes, and the CA extracts are immobilised by simple drop casting on nitrocellulose paper, making use of their extraordinary capacity for biomolecule immobilisation.^28^

**TABLE:** Electrochemical immunosensing for cutaneous leishmaniasis

Supportw	Electrode	Bioreceptor	Bioreceptor immobilisation	Detection method	Limit of detection	Samples	Reference
Paper Whatman N° 1	PGE^*b*^	*Leishmania (V.) braziliensis*, crude antigenic extracts	Nitrocellulose paper	EIS^*e*^	1/60 dilution	Human serum	Our platform
PAMAM^*a*^ /proteoliposomes	Au-IDE^*c*^	*L. amazonensis*, crude antigenic extracts	Proteoliposomes	EIS	1 x 10^-8^ g/mL	Mice blood	^28^
Cellulose acetate	Au	*L. (V.) braziliensis* epitope PSA-38S	PA6^*d*^ /chitosan (60/40)	CM^*f*^	1.6 x 10^-12^ pg/mL	Human serum	^29^

*a*: polyamidoamine generation 4 dendrimers; *b*: pencil graphite electrode; *c*: gold interdigitated electrodes; *d*: polyamide 6; *e*: electrochemical impedance spectroscopy; *f*: capacitance measurements.

Finally, of all the works in this area, we found only one alternative label-free electrochemical immunosensor for the rapid diagnosis of visceral leishmaniasis using *L. (V.) braziliensis* antigens. Here, the authors immobilise a peptide-based probe of the promastigote surface antigen (PSA-38S) on electrospun polyamide-6 (PA6)/chitosan nanofibers, with good results.^38^ In this sense, it is important to emphasise again the technological simplicity of our device and that, in our case, we worked with real samples and not with synthetic reagents. Thus, our device uses CA extracts from *L. (V.) braziliensis* promastigotes as well as sera samples from patients infected with the same parasite.

Historically, serological methods have been important diagnostic tools for various infectious diseases owing to their low cost and technical simplicity, such as making a diagnosis using a single drop of blood. Detection of anti-*Leishmania* AB in patient sera is particularly relevant in cases where it is difficult to obtain adequate biological material from lesions or because sample extraction is painful due to secondary bacterial infection.^6^

As in other works,^39^ ^,^ ^40^ our device requires a second stage of testing to establish its diagnostic efficacy. Adjustments of conditions, detection limits, predictive values, sensitivity, and specificity are necessary. The determination of these statistical indicators and the improvement of the reproducibility problems of our platform will allow, in the future, solid foundations for the diagnosis of ATL in both dogs and humans in our region.
